# Delta Neutrophil Index and Other Hematologic Parameters in Acute Exacerbations of COPD: A Retrospective Study

**DOI:** 10.1155/carj/3647362

**Published:** 2025-08-28

**Authors:** Burcu Akkok, Evrim Gulderen Kuscu, Hatice Sahin

**Affiliations:** ^1^Department of Pulmonology, Sutcu Imam University Faculty of Medicine, Kahramanmaras, Turkey; ^2^Department of Infectious Disease and Clinical Microbiology, Sutcu Imam University Faculty of Medicine, Kahramanmaraş, Turkey

**Keywords:** acute exacerbation, chronic obstructive pulmonary disease, delta neutrophil index, mortality

## Abstract

**Background:** Chronic obstructive pulmonary disease (COPD) is an increasing cause of morbidity and mortality worldwide, and acute exacerbations are the major health issues in COPD patients. In this study, we aimed to investigate the role of the delta neutrophil index (DNI) with other hematologic parameters in managing and guiding COPD patients admitted with acute exacerbations.

**Methods:** In this retrospective study, COPD patients treated internally in pulmonology clinic, intensive care unit, and anesthesiology and reanimation unit with acute exacerbation between May 2021 and December 2023 were investigated. Records from daily visits were evaluated retrospectively. Patients were divided into two groups according to the causative organism: bacterial or nonbacterial.

**Results:** Patients with cardiac failure were found to have significantly higher median DNI values (*p* : 0.026), whereas patients with other comorbidities that were not individually recorded have substantially lower median DNI values (*p* : 0.026). White blood cell (WBC), neutrophil, immature granulocyte values (both absolute value and percent), thrombocyte, platelet–lymphocyte ratio (PLR), neutrophil–lymphocyte ratio (NLR), C-reactive protein (CRP), procalcitonin, positive blood culture, positive systemic inflammatory response syndrome (SIRS) criteria, and sepsis were significantly higher in patients with bacterial acute exacerbation. Hospitalization duration was also significantly longer in the same group (*p* :  0.006). No statistically significant correlation was found between median DNI values and early mortality rate (within 28 days), readmission within 30 days and 6 months.

**Conclusion:** In this study, we have shown that the serum procalcitonin level, WBC, NLR, and PLR measurement can be used to distinguish bacterial and nonbacterial COPD exacerbations. The DNI revealed no prognostic predictive value regarding early mortality, mechanic ventilation need, or readmission in 30 days and 6 months.

## 1. Introduction

Chronic obstructive pulmonary disease (COPD) is an increasing cause of morbidity and mortality worldwide. Disease courses exist in a wide range with some patients suffering from minimal symptoms but having a high degree of obstruction, while another group of patients have better lung function but have severe symptoms. The main therapeutic objectives are minimizing symptoms to obtain an improvement in exercise tolerance and reduce acute exacerbation risk [1].

Acute exacerbations are major health issues affecting the quality of life in COPD patients, which have a negative impact on disease progression leading to a consequential increase in hospitalization and mortality [2–4]. The study by Guerreno et al. demonstrated an association between readmission for exacerbations and a progressive increase in long-term mortality [5]. In exacerbations, systemic inflammation increases as well as airway inflammation and is mostly associated with bacterial or viral infective triggers [4]. Increased frequency of exacerbations also causes a significant decline in forced expiratory volume in 1 s (FEV1) [6], which consequentially increases disease severity and mortality [7]. Community-acquired pneumonia (CAP) is a frequent companion in these patients [8] and is a major cause of exacerbations and longer hospitalization periods [9]. Older age, disease severity, and corticosteroid consumption are determined as some of the predisposing factors for CAP in COPD patients [9, 10]. Some previous studies stated that about 20% of patients were readmitted due to acute exacerbation within 30 days after discharge following inpatient treatment [11, 12]. However, actual literature data are insufficient to provide any clinical or biological marker for the prediction of patients with a higher risk of readmission due to acute exacerbations. Some indexes derived from hematological parameters such as neutrophil–lymphocyte ratio (NLR) and platelet–lymphocyte ratio (PLR) are suggested as C-reactive protein (CRP) like biomarkers for diagnosis and evaluation of acute exacerbations in COPD patients [13].

The delta neutrophil index (DNI) is the immature granulocyte fraction obtained via a blood cell analyzer (ADVIA 2120; Siemens Healthcare Diagnostics, Deerfield, III). It is calculated by subtracting the fraction of mature polymorphonuclear leukocytes from the total amount of myeloperoxidase reactive cells [14, 15]. DNI is cost-effective in clinical practice while it can easily be calculated and reported. Some recent systematic reviews and meta-analyses showed the prognostic value of DNI in inflammatory situations and sepsis [16]. In addition, intensive care unit hospitalizations and mortality were found to be higher in neutrophil-dominated COPD exacerbations; therefore, hematologic parameters to be examined at the beginning of treatment have gained importance in determining the prognosis [17]. Therefore, DNI can be a useful tool to predict the prognosis of COPD patients, especially with pneumonia and exacerbations [18].

The aim of this study was to investigate the relationship between DNI and other hematologic markers in differentiating bacterial and nonbacterial exacerbations and prognostic implications.

## 2. Methods

### 2.1. Subjects

In this retrospective study, COPD patients treated internally in Kahramanmaraş Sütçü İmam University Faculty of Medicine Hospital pulmonology clinic, intensive care unit, and anesthesiology and reanimation unit with acute exacerbation between May 2021 and December 2023 were investigated.

The study was approved by the Local Ethics Committee of the Medical Faculty of the Sutcu Imam University (11.10.2022/04). All procedures were performed in terms of the ethical standards of the institutional research committee in alliance with the 1964 Helsinki Declaration and its later amendments. Informed consent was waived owing to the retrospective nature of the study. In addition to the GOLD and Rome criteria, the presence of any of the following clinical conditions was accepted [19].

### 2.2. Admission Criteria

Acute exacerbation criteria were as follows:

1. Failure to respond to initial medical treatment,
2. Severe symptoms (resting dyspnea, coughs, sputum production, confusion, or drowsiness),
3. Newly started cyanosis, peripheral edema,
4. Respiratory rates ≥ 30 breaths/min, oxygen saturation ≤ 90%,
5. Respiratory failure (accompanied by mental changes and respiratory muscles used) [20].

### 2.3. Exclusion Criteria

Patients with

1. Cardiac valvular disease
2. Medical history of myocardial infarct
3. Medical history of cerebral infarct
4. Cerebral hemorrhage
5. Untreated malignancy
6. Renal failure having hemodialysis
7. Missing data.

### 2.4. Data Collection and Verification

Records from daily visits were evaluated retrospectively. Patients were divided into two groups according to the causative organism: bacterial or nonbacterial. Patients with cough, at least one lower respiratory tract symptom (shortness of breath, phlegm, chest pain, and side pain), increased sputum purulence, new focal signs in thorax physical exam, and at least one systemic symptom (fever ≥ 38°C, pain, and sweating) were chosen for bacterially caused exacerbations. Infectious agents isolated from sputum, bronchoalveolar lavage, and blood samples were reported. Sputum samples were accepted only if > 25 leucocytes, and < 10 epithelial cells were detected per high power field [21]. White blood cell (WBC) count, DNI, NLR, PLR, hemoglobin, CRP, and procalcitonin (PCT) values were obtained from the blood samples taken within the first hour after admission. Complete blood count (CBC) is performed via Sysmax XN_3000 [Sysmax Corp., Kobe, Japan].

Patients were investigated regarding if they met the criteria of systemic inflammatory response syndrome (SIRS) or sepsis according to physical exam and laboratory findings.

### 2.5. Statistical Analysis

SPSS 25.0 (IBM Corporation, Armonk, New York, United States) and MedCalc 14 (Acacialaan 22, B-8400 Ostend, Belgium) programs were used in the analysis of variables. The Shapiro–Wilk Francia test was used to assess the normal distribution conformity of the data, while the Levene test was used to assess the homogeneity of variance. In the comparison of two independent groups according to quantitative variables, the Mann–Whitney *U* test was used with Monte Carlo results, and the Independent Samples *t* Test was used with bootstrap results. In the comparison of categorical variables, the Pearson chi-square test, Fisher Freeman Halton test, and Fisher exact test were used, and post hoc analyses were done with the Benjamini–Hochberg test. Spearman rho correlation test was used for the comparison of quantitative variables. DNI and acute exacerbation causes are classified via a calculated cut-off value. To determine the relationship between the cut-off-based classification and actual classification sensitivity, specificity, positive identification rate (positive predictivity), and negative identification rate (negative predictivity) rates are analyzed with ROC (receiver operating curve) analysis. Cut-off values were derived using the Youden Index. A logistic regression analysis was performed using the backward elimination method to determine the relationship between acute exacerbation cause and explanatory variables. Variables with a *p* value < 0.10 in univariate analysis were considered for inclusion in the multivariable model. Quantitative variables were expressed as the mean (standard deviation) and median (minimum/maximum) in the tables, while categorical variables were shown as *n* (%). Variables were examined at a 95% confidence level, and *p* value less than 0.05 was accepted as significant.

## 3. Results

Totally 146 patients suitable for inclusion criteria who were admitted with acute exacerbations were enrolled in the study. The mean age was 73.6 (42–104) year-old, and 107 patients (73.3%) were male.

The demographic characteristics, symptoms and clinical findings, comorbidities, and laboratory data of patients are shown in Table 1.

When we investigated the relationship between comorbidities and DNI values; patients with cardiac failure were found to have significantly higher median DNI values (0.73, *p* : 0.026), whereas patients with other comorbidities that were not individually recorded have significantly lower median DNI values (0.67, *p* : 0.026) (Table 2).

No statistically significant correlation was found between median DNI values and early mortality rate (within 28 days), and readmission within 30 days and 6 months (Table 2).

When the correlation of DNI values with hematologic variables was analyzed (Table 3), WBC, neutrophil, and immature granulocyte values (both absolute value and percent) were found statistically significant (*p* : 0.001,  *p* : 0.005,  *p* < 0.001, respectively).

Laboratory and microbiologic findings, radiologic infiltration, and comorbidities were also analyzed regarding the cause of acute exacerbation (Table 4). WBC, neutrophil, immature granulocyte values (both absolute value and percent), thrombocyte, PLR, NLR, CRP, PCT, positive blood culture, positive SIRS criteria, and sepsis were found significantly higher in patients with bacterially caused acute exacerbation. Hospitalization duration was also significantly longer in the same group (*p* : 0.006). On the contrary, in patients with cardiac failure, nonbacterial cause was significantly higher (*p* : 0.001). Radiological images of patients with nonbacterial acute exacerbation mostly revealed no infiltration or unilobar infiltrations (*p*:0.004 and *p*:0.037). Mechanic ventilation necessity was also significantly higher in patients who were admitted with acute exacerbation due to a bacterial cause (*p*:0.004). Early mortality and readmission within 30 days or 6 months were not significantly different between the two groups.

Streptococcus pneumoniae and Enterococcus subspecies were the most frequently isolated pathogens (17.5%); Escherichia coli (12.5%), Pseudomonas subspecies (12.5%), Haemophilus Influenza (7.5%), Acinetobacter (7.5%), Klebsiella pneumoniae (5.0%), and other pathogens (5%) were isolated (Table 1).

**Figure 1:**
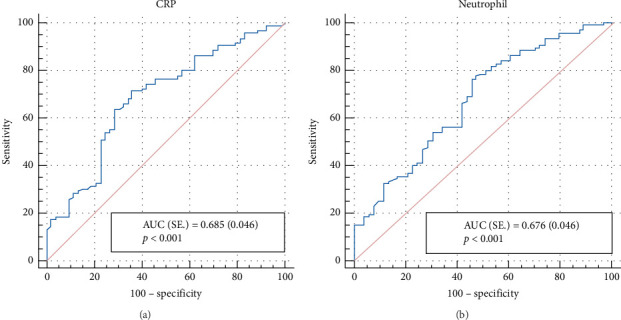
(a) Graphical demonstration of sensitivity, specificity, and AUC for CRP. (b) Graphical demonstration of sensitivity, specificity, and AUC for neutrophil.

We also sought for an optimal cut-off value for hematologic parameters to differentiate the acute exacerbation cause (bacterial or nonbacterial) using the ROC curve (Table 5). The calculated optimal cut-off value for WBC is ≤ 10.58, which has a 59.4% sensitivity rate and the specificity rate was 67.92%. The positive and negative identification rates were 76.4% and 48.6%, respectively, and the area under the curve (AUC) was calculated as 0.655 ± 0.047. This cut-off value statistically sufficiently distinguishes the cause of attacks (*p*=0.001). As the interpretation, it can be stated that if WBC≤ 10.58, then the nonbacterially stimulated exacerbation rate is increased. Cut-off values obtained for neutrophil, NLR, thrombocyte, PLR, immature granulocyte absolute and percent values, CRP, PCT, and hospitalization duration (Days) were statistically significant for distinguishing the acute exacerbation cause (*p* : 0.032 − *p* < 0.001). The AUC values for all these variables revealed adequate accuracy. Sensitivity, specificity, and AUC values for CRP and neutrophil are shown in Figures 1(a) and 1(b).

Categoric variables statistically significant for exacerbation cause (Table 4) and significant variables categorized according to cut-off values (Table 5) were analyzed to predict acute exacerbation cause. The presence of sepsis was found to be the major variable with the odds ratio of 5.53 [1.517–20.156]. In the absence of sepsis, acute exacerbation with any cause other than bacteria is 5.53 times more. The rate of exacerbation due to bacterial causes was 3.139 times higher in patients with CRP > 31.8 compared with patients having CRP ≤ 31.8, which was statistically significant [95% CI: 1.397–7.054] (Table 6).

## 4. Discussion

The DNI is calculated as the difference between leukocyte differentials measured in the cytochemical myeloperoxidase channel and those measured in the nuclear lobularity channel, identifying the fraction of circulating immature granulocytes [22, 23]. Prior studies have shown that immature granulocyte precursors are more reliable markers of infection than band neutrophils [24]. Furthermore, DNI has demonstrated a superior diagnostic value compared to the total WBC count and absolute neutrophil count in conditions such as sepsis and septic shock [23]. A recent systematic review and meta-analysis also confirmed the association between elevated DNI and mortality in adult patients with sepsis [15].

Beyond sepsis, DNI has been explored as a diagnostic and prognostic biomarker in other infectious diseases, including pneumonia, tuberculosis, and acute prostatitis [25–27]. However, its application in the context of COPD, particularly during acute exacerbations, remains limited. One observational study reported that 30-day readmissions in patients with acute exacerbation of COPD (AECOPD) are linked to increased mortality [5]. Park et al. further demonstrated that patients with DNI ≥ 3.5% who were readmitted within 30 days had the lowest cumulative survival rates. Despite this, our study did not find a significant correlation between DNI values and early mortality in AECOPD patients, suggesting the need for further investigation to define the prognostic utility of DNI in this patient population.

Our findings diverge from studies in other clinical scenarios. For instance, Han et al. found significantly lower DNI values in patients who survived cardiac arrest compared to those who did not (*p*=0.005) [28]. Additionally, Park et al.'s meta-analysis of 12 studies found a pooled sensitivity of 0.67 and specificity of 0.94 for infection prediction using DNI, and as a prognostic indicator of death in infected patients, DNI yielded an AUC of 0.89, with a pooled sensitivity of 0.70 and specificity of 0.78 [29].

PCT is a frequently investigated biomarker to distinguish bacterial and nonbacterial acute COPD exacerbation. In their recent study, Daubin et al. found significantly higher PCT levels in patients with bacterial infection compared to patients without any documented pathogens (*p*  <  0.001). However, despite higher levels, authors stated that PCT had a poor accuracy in distinguishing bacterial and nonbacterial infection [30]. Similarly, van de Geijin et al. evaluated some novel laboratory tests to discriminate bacterial and nonbacterial COPD exacerbations. They found that basophil percentage (CytoDiff) has a superior AUC (0.800). According to their results, with the cutoff resulting ≥ 90% sensitivity, NLR (AUC: 0.755) and CD4-positive T cells (Leukoflow, AUC: 0.747) have the highest specificity (57%). When leukocyte populations and PCT were added to CRP in regression models, the specificity increased compared to CRP alone (71% or 73% vs. 39%). Authors concluded that none of these tests were sufficiently accurate alone to predict bacterial exacerbations; therefore, combination of CRP with several other parameters is suggested to improve this [31].

In a study that compared several parameters as a diagnostic marker for bacterial exacerbations, mean PCT levels were found to be significantly higher in patients with positive sputum cultures and the cut-off values for PCT, CRP, and the N/L ratio were calculated as 0.40 ng/mL, 91.50 mg/L, and 11.5, respectively. The AUC value of PCT for predicting bacterial infection was significantly better compared to CRP or N/L ratio (*p*=0.042). It is also stated that PCT was not found to be so reliable in predicting bacterial exacerbations, while sensitivity and specificity were less than 80% [32].

On the contrary, the results of another recent study revealed significantly higher mean serum PCT levels in patients with bacterial COPD exacerbations. With a 0.9 ng/mL cutoff, PCT had 100% sensitivity and 76.9% specificity in predicting bacterial exacerbations.

The authors concluded that serum PCT levels can be utilized as an appropriate biomarker to diagnose bacterial exacerbations in COPD [33].

In line with the above studies, our results revealed significantly higher serum PCT levels in patients with bacterial COPD exacerbations.

Pneumonic COPD exacerbations which were defined as pneumonic infiltrates on chest x-ray and CRP value of ≥ 40 mg/L were found to increase hospitalization duration and in-hospital morbidity [34].

In our study, multilobar pulmonary infiltrations and hospitalization duration were both significantly higher in patients with bacterial COPD exacerbations.

Antibiotic prescription in these patients is another challenging issue. Ruiz-González et al. created a scoring system including CRP and blood neutrophil count to distinguish bacterial and nonbacterial acute exacerbations. Interpreting the result of their study, the authors concluded that this is a simple scoring system, which can guide clinicians in terms of prescribing antibiotics in acute COPD exacerbations [35].

The presence of sepsis was found to be the major variable regarding the cause of COPD exacerbations in our study and acute exacerbation with any cause other than bacteria was calculated as 5.53 times more in the absence of sepsis.

Our study has several limitations. First, the number of patients and events was relatively limited, which may reduce the statistical power and generalizability of the findings. Second, the retrospective nature of the study limited our ability to control for potential confounders, including prior antibiotic use, comorbidities, and baseline functional status. Inflammatory markers such as PCT, WBC, and DNI may also be influenced by age-related changes in the immune system, which should be considered when interpreting results. Furthermore, although ROC analysis was used to evaluate the diagnostic utility of various markers, confidence intervals for AUC values were not reported, and external validation was not performed. Additionally, the study did not evaluate multicollinearity among variables, which may influence regression model validity. Furthermore, GOLD staging and treatment history were not included due to retrospective data limitations. Lastly, ventilatory support subgroups were not categorized in the analysis, which limits interpretation of related outcomes.

## 5. Conclusions

In this study, we demonstrated that serum PCT, WBC count, NLR, and PLR—parameters that are readily available in routine blood tests—may provide supportive information in differentiating bacterial from nonbacterial causes of acute COPD exacerbations. However, their diagnostic performance was modest, and they should not be used in isolation.

The DNI, while higher in patients with bacterial infections, showed no prognostic value for predicting in-hospital mortality, need for mechanical ventilation, or readmission within 30 days or 6 months.

Therefore, DNI should not be considered a reliable prognostic biomarker in AECOPD. Future prospective, multicenter studies with larger patient populations and adjustment for clinical confounders are warranted to validate these findings and explore their utility in clinical decision-making.

## Figures and Tables

**Table 1:** Demographic and clinical features of the patients.

Variables		*n* (%)	Mean (SD)		Median (min/max)
*Demographics*					
Gender (male)		107 (73.3%)	—		—
Age		—	73.62 (10.77)		74.5 (42/104)
*Hospitalization*					
Service		39 (26.7%)	—		—
Intensive care		24 (16.4%)	—		—
Service + intensive care		83 (56.8%)	—		—
Hospitalization duration (days)		—	9.79 (7.14)		8 (1/45)

**Variables**		***n* (%)**	**Variables**		***n* (%)**

*Comorbidities*			*Microorganism (blood culture)*		
Hypertension		75 (51.4)	Staphylococcus epidermidis		6 (26.1)
Ischemic heart disease		33 (22.6)	Enterecoc subspecies		10 (43.5)
Cardiac failure		37 (25.3)	Escherichia coli		3 (13)
Diabetes		42 (28.8)	Staphylococcus aureus		2 (8.7)
Cerebrovascular disease		7 (4.8)	Other		2 (8.7)
Other		63 (43.2)	*Cultures and infection findings*		
*Symptoms*			Positive SIRS criteria		42 (28.8)
Dyspnea		145 (99.3)	Sepsis		13 (8.9)
Cough		130 (89)	Positive sputum/lavage culture		40 (60.6)
Sputum		125 (85.6)	Blood culture (positive)		23 (19.3)
Confusion		10 (6.8)	*Radiology*		
Lethargy		4 (2.7)	No infiltration		67 (45.9)
Edema		48 (32.9)	Unilobular		58 (39.7)
Cyanosis		109 (74.7)	Multilobular		14 (9.6)
*Positive microorganism*			Ground glass		3 (2.1)
Streptococcus pneumonia		7 (17.5)	Multiple infiltrations		4 (2.7)
Haemophylus influenza		3 (7.5)	*Mechanic ventilation (MV)*		
Pseudomonans subspecies		5 (12.5)	None		74 (50.7)
Staphylococcus subspecies		1 (2.5)	Noninvasive MV		58 (39.7)
Serratia subspecies		3 (7.5)	MV		14 (9.6)
Stenotroph		2 (5)	*Treatments*		
Klebsialla pneumonia		2 (5)	Oxygen replacement		144 (98.6)
Acinetobacter		3 (7.5)	Bronchodilator		144 (98.6)
Enterecoc subspecies		7 (17.5)	Steroids		123 (84.2)
Escherichia coli		5 (12.5)	Antibiotics		144 (98.6)
Other		2 (5)	Antiviral		3 (2.1)
*Outcomes*			*Outcomes*		
Early mortality		138 (94.5)	Readmission within 30 days		27 (18.5)
Cause of acute exacerbation (other)		93 (63.7)	Readmission within 6 months		44 (32.4)

**Variables**	**Mean (SD)**	**Median (min/max)**	**Variables**	**Mean (SD)**	**Median (min/max)**

*Lab values*			*Lab values*		
Pulse	81.36 (8.57)	84 (50/97)	Hb (g/dL)	12.95 (2.59)	12.9 (7.6/20.6)
Wbc (× 10^3^)	12.04 (6.38)	10.6 (3.08/53.33)	Htc (%)	42.19 (8.07)	42.2 (26.3/71.5)
Neu (× 10^3^)	9.79 (6.15)	8.37 (2.36/51.27)	IG	0.13 (0.24)	0.06 (0.01/1.98)
Lym (× 10^3^)	1.23 (0.77)	1.13 (0.19/4.98)	IG%	0.88 (1.09)	0.5 (0.04/7.2)
Neu/Lym	12.95 (15.7)	7.37 (1.06/119.23)	DNI (× 10^−5^)	1.12 (1.41)	0.69 (0.21/9.03)
PLT (× 10^4^)	24.41 (8.89)	23.15 (8.8/52.5)	CRP (mg/L)	63.92 (70.17)	31.65 (3/300)
PLT/Lym	306.6 (283.1)	204.45 (44.9/1660.7)	PCT (ug/L)	0.54 (1.12)	0.14 (0.01/5.8)

**Table 2:** Comparison of median DNI values according to comorbidities, symptoms, and treatment.

		**DNI (×**10^−5^**)**			**p**
		**Median (min/max)**		**Median (min/max)**	

Gender	Female/male	0.69 (0.23/7.37)	/	0.71 (0.21/9.03)	0.606^u^
Hypertension	Yes/No	0.69 (0.21/5.81)	/	0.73 (0.23/9.03)	0.335^u^
Ischemic heart disease	Yes/No	0.69 (0.21/9.03)	/	0.72 (0.23/4.29)	0.716^u^
Cardiac failure	Yes/No	0.73 (0.21/9.03)	/	0.61 (0.23/7.37)	0.026^u^
Diabetes	Yes/No	0.67 (0.21/9.03)	/	0.74 (0.26/7.37)	0.117^u^
Cerebrovascular disease	Yes/No	0.69 (0.21/9.03)	/	1.41 (0.42/2.39)	0.076^u^
Other	Yes/No	0.67 (0.21/7.37)	/	0.77 (0.23/9.03)	0.026^u^
Dyspnea	Yes/No	0.47 (0.47/0.47)	/	0.69 (0.21/9.03)	0.436^u^
Cough	Yes/No	0.63 (0.29/7.1)	/	0.69 (0.21/9.03)	0.431^u^
Sputum	Yes/No	0.76 (0.29/7.37)	/	0.69 (0.21/9.03)	0.937^u^
Confusion	Yes/No	0.7 (0.21/9.03)	/	0.68 (0.29/1.68)	0.642^u^
Lethargy	Yes/No	0.69 (0.21/9.03)	/	0.62 (0.29/1.41)	0.583^u^
Cyanosis	Yes/No	0.72 (0.21/7.37)	/	0.69 (0.23/9.03)	0.884^u^
Positive sputum/lavage culture	Yes/No	0.65 (0.23/7.37)	/	0.76 (0.21/5.81)	0.189^u^
Positive blood culture	Yes/No	0.67 (0.23/9.03)	/	0.76 (0.23/2.51)	0.084^u^
Positive SIRS criteria	Yes/No	0.69 (0.21/9.03)	/	0.71 (0.28/5.2)	0.715^u^

		**Mean ** **(SD)**		**Mean (SD)**	

Edema	Yes/No	1.01 (1.09)	/	1.37 (1.89)	0.229^t^
Sepsis	Yes/No	1.11 (1.44)	/	1.25 (0.95)	0.634^t^

		**Median (min/max)**		**Median (min/max)**	

Radiology	No infiltration	0.62 (0.23/9.03)		—	0.132^k^
	Unilobar	0.76 (0.28/7.37)		—	
	Multilobar	0.98 (0.23/7.37)		—	
	Ground glass	0.51 (0.44/0.76)		—	
	Multiple infiltrations	0.76 (0.21/1)		—	
Oxygen replacement	Yes/No	0.78 (0.23/1.33)	/	0.69 (0.21/9.03)	0.689^u^
Bronchodilator	Yes/No	0.43 (0.36/0.5)	/	0.7 (0.21/9.03)	0.112^u^
Steroids	Yes/No	0.69 (0.21/7.1)	/	0.69 (0.23/9.03)	0.814^u^
Antibiotics	Yes/No	0.55 (0.38/0.72)	/	0.69 (0.21/9.03)	0.418^u^
Antiviral	Yes/No	0.69 (0.21/9.03)	/	0.63 (0.59/1.13)	0.928^u^

		**Mean (SD)**		**Mean (SD)**	

Mechanical ventilation (MV)	None	1.11 (1.46)		—	0.976^M^
	Noninvasive MV	1.15 (1.46)		—	
	MV	1.08 (0.9)		—	

		**Median (min/max)**		**Median (min/max)**	

Hospitalization	Service	0.72 (0.23/7.37)		—	0.609^k^
	Intensive care	1.03 (0.39/9.03)		—	
	Service + intensive care	0.67 (0.21/4.29)		—	
Early mortality (within 28 days)	No/Yes	1.01 (0.39/5.2)	/	0.69 (0.21/9.03)	0.217^u^
Cause of acute exacerbation	Bacterial/Other	0.76 (0.21/7.1)	/	0.67 (0.23/9.03)	0.191^u^
Readmission within 30 days	No/Yes	0.71 (0.23/9.03)	/	0.67 (0.21/2.39)	0.095^u^
Readmission within 6 months	No/Yes	0.75 (0.23/9.03)	/	0.68 (0.21/2.11)	0.080^u^

*Note:* min: Minimum, max: Maximum.

Abbreviation: SD, standard deviation.

^k^Kruskal–Wallis H test (Monte Carlo).

^M^One-way ANOVA (bootstrap).

^t^Independent samples *t* test (bootstrap).

^u^Mann–Whitney *U* test (Monte Carlo).

**Table 3:** Correlation DNI with clinical and laboratory variables.

	DNI (× 10^−5^)	
	*r*	*p*
Age	0.061	0.462
Pulse	0.081	0.330
WBC (× 10^3^)	0.271	**0.001**
Neutrophil (× 10^3^)	0.231	**0.005**
Lymphocyte (× 10^3^)	0.148	0.074
Neutrophil/Lymphocyte (× 10^3^)	0.032	0.705
Thrombocyte (× 10^4^)	0.109	0.189
Platelet/lymphocyte	−0.062	0.456
Hb	−0.101	0.224
Htc	−0.133	0.109
Immature granulocyte (Ig)	0.841	**< 0.001**
Ig (%)	0.932	**< 0.001**
CRP	0.092	0.269
Procalcitonin	0.082	0.327
Hospitalization duration (days)	0.050	0.548

*Note:* Pearson correlation test and Spearman rho test; *r*: correlation coefficient. The bold values indicate the significance threshold of the *p* value and values above it.

**Table 4:** Comparison of clinical and laboratory findings in patients with bacterial and nonbacterial acute COPD exacerbations.

	Cause of acute exacerbation (bacterial)	Cause of acute exacerbation (other)	*p*
	(*n* = 53)	(*n* = 93)	
Age, mean (SD)	72.06 (10.56)	74.52 (10.83)	0.204^t^
Gender (male), *n* (%)	39 (73.6)	68 (73.1)	0.999^c^
Hypertension, *n* (%)	22 (41.5)	53 (57)	0.086^c^
Ischemic heart disease, *n* (%)	11 (20.8)	22 (23.7)	0.817^c^
Cardiac failure, *n* (%)	5 (9.4)	32 (34.4)	0.001^c^
Diabetes, *n* (%)	11 (20.8)	31 (33.3)	0.130^c^
Cerebrovascular disease, *n* (%)	4 (7.5)	3 (3.2)	0.256^f^
Other, *n* (%)	26 (49.1)	37 (39.8)	0.301^c^
Dyspnea, *n* (%)	53 (100)	92 (98.9)	0.999^f^
Cough, *n* (%)	49 (92.5)	81 (87.1)	0.414^c^
Sputum, *n* (%)	45 (84.9)	80 (86)	0.999^c^
Confusion, *n* (%)	3 (5.7)	7 (7.5)	0.748^f^
Lethargy, *n* (%)	1 (1.9)	3 (3.2)	0.999^f^
Edema, *n* (%)	14 (26.4)	34 (36.6)	0.272^c^
Cyanosis, *n* (%)	37 (69.8)	72 (77.4)	0.328^c^
Pulse, median (min/max)	84 (50/96)	83 (60/97)	0.746^u^
WBC (× 10^3^), median (min/max)	12.15 (4.87/53.33)	9.96 (3.08/33.76)	0.001^u^
Neutrophil (× 10^3^), median (min/max)	10 (4.28/51.27)	7.75 (2.36/29.89)	0.001^u^
Lymphocyte (× 10^3^), median (min/max)	0.93 (0.19/4.98)	1.18 (0.24/3.89)	0.134^u^
Neutrophil/Lymphocyte, median (min/max)	9.63 (1.06/119.23)	6.22 (1.58/47.83)	0.001^u^
Thrombocyte (× 10^4^), median (min/max)	25.3 (8.8/52.5)	20.5 (9.4/43.2)	0.012^u^
Platelet–lymphocyte ratio, median (min/max)	253.04 (56.64/1660.71)	180.34 (44.99/1355.17)	0.015^u^
Hb, mean (SD)	12.7 (2.68)	13.09 (2.54)	0.391^t^
Htc, mean (SD)	41.15 (7.75)	42.79 (8.23)	0.231^t^
Immature granulocyte (Ig), median (min/max)	0.09 (0.01/1.98)	0.05 (0.01/1.16)	0.002^u^
Ig%, median (min/max)	0.6 (0.2/5.9)	0.5 (0.04/7.2)	0.032^u^
DNI (× 10^−5^), median (min/max)	0.76 (0.21/7.1)	0.67 (0.23/9.03)	0.187^u^
CRP, median (min/max)	72.9 (3.3/300)	22.6 (3/300)	< 0.001^u^
Procalcitonin, median (min/max)	0.19 (0.01/5.8)	0.1 (0.01/3.86)	0.001^u^
Positive blood culture, *n* (%)	17 (63)	6 (6.5)	< 0.001^c^
Microorganism, *n* (%)			0.530^ff^
Staphylococcus epidermidis	5 (29.4)	1 (16.7)	
Enterococcus subspecies	6 (35.3)	4 (66.7)	
Escherichia coli	3 (17.6)	0 (0)	
Staphylococcus aureus	1 (5.9)	1 (16.7)	
Other	2 (11.8)	0 (0)	
Positive SIRS criteria, *n* (%)	28 (52.8)	14 (15.1)	< 0.001^c^
Sepsis, *n* (%)	10 (18.9)	3 (3.2)	0.002^f^
Radiology, *n* (%)			0.042^ff^
No infiltration	16 (30.2)	51 (54.8)	0.004
Unilobular	27 (50.9)	31 (33.3)	0.037
Multilobular	7 (13.2)	7 (7.5)	ns
Ground glass	1 (1.9)	2 (2.2)	ns
Multiple infiltrations	2 (3.8)	2 (2.2)	ns
Oxygen replacement, *n* (%)	52 (98.1)	92 (98.9)	0.999^f^
Bronchodilator, *n* (%)	51 (96.2)	93 (100)	0.130^f^
Steroids, *n* (%)	42 (79.2)	81 (87.1)	0.241^c^
Antibiotics, *n* (%)	53 (100)	91 (97.8)	0.534^f^
Antiviral, *n* (%)	2 (3.8)	1 (1.1)	0.298^f^
Mechanical ventilation (MV), *n* (%)			0.016^c^
None	25 (47.2)	49 (52.7)	ns
Noninvasive MV	18 (34)	40 (43)	ns
MV	10 (18.9)	4 (4.3)	0.004
Hospitalization, *n* (%)			0.265^c^
Service	10 (18.9)	29 (31.2)	
Intensive care	9 (17)	15 (16.1)	
Service + intensive care	34 (64.2)	49 (52.7)	
Hospitalization duration (days), median (min/max)	10 (2/45)	7 (1/34)	0.006^u^
Early mortality, *n* (%)	50 (94.3)	88 (94.6)	0.999^f^
Readmission within 30 days, *n* (%)	10 (18.9)	17 (18.3)	0.999^c^
Readmission within 6 months, *n* (%)	13 (26.5)	31 (35.6)	0.341^c^

*Note:* min: minimum, max: maximum.

Abbreviation: SD, standard deviation.

^c^Pearson chi-square test (Monte Carlo); post hoc: Benjamini–Hochberg test.

^f^Fisher exact test (Monte Carlo).

^ff^Fisher Freeman Halton test (Monte Carlo); post hoc: Benjamini–Hochberg test.

^t^Independent samples *t* test (bootstrap).

^u^Mann–Whitney *U* test (Monte Carlo).

**Table 5:** Sensitivity, specificity, and AUC of laboratory variables regarding the prediction of exacerbation cause.

Cause of exacerbation (other)	Cut off	Sensitivity	Specificity	+PV	−PV	AUC ± SE.	P Değeri
WBC (× 10^3^)	≤ 10.58	59.14	67.92	76.4	48.6	0.655 ± 0.047	0.001
Neutrophil (× 10^3^)	≤ 9.76	76.34	54.72	74.7	56.9	0.676 ± 0.046	< 0.001
Neutrophil/lymphocyte	≤ 8.478	65.59	60.38	74.4	50.0	0.660 ± 0.048	0.001
Thrombocyte (× 10^4^)	≤ 21.1	52.69	73.58	77.8	47.0	0.625 ± 0.049	0.011
Platelet/lymphocyte	≤ 226.415	62.37	60.38	73.4	47.8	0.622 ± 0.049	0.013
Immature granulocyte (Ig)	≤ 0.1	79.57	45.28	71.8	55.8	0.650 ± 0.048	0.002
Ig%	≤ 0.8	82.8	35.82	69.4	54.3	0.607 ± 0.050	0.032
CRP	≤ 31.8	63.44	71.7	79.7	52.8	0.685 ± 0.046	< 0.001
Procalcitonin	≤ 0.1	53.76	73.58	78.1	47.6	0.660 ± 0.047	0.001
Hospitalization duration (days)	≤ 9	70.97	56.6	74.2	52.6	0.639 ± 0.048	0.004

*Note:* ROC: receiver operating curve analysis (Honley & Mc Nell–Youden index J), AUC: area under the ROC curve, +PV: positive predictive value, and −PV: negative predictive value.

Abbreviation: SE, standard error.

**Table 6:** Efficiency of clinical variables regarding the prediction of exacerbation cause.

Reference groups: cause of exacerbation (other)	B (SE)	*p* value	Odds ratio (95% CI)
Cardiac failure	1.36 (0.53)	0.010	3.907 [1.391–10.972]
Positive SIRS criteria	1.65 (0.51)	0.001	5.208 [1.926–14.082]
Sepsis	1.71 (0.66)	0.010	5.53 [1.517–20.156]
CRP	1.14 (0.41)	0.006	3.139 [1.397–7.054]
Accuracy rates	Bacterial: 73.6	Other: 82.8	General: 79.5

*Note:* Multiple logistic regression (method = backward stepwise (Wald)); B: regression coefficients.

Abbreviations: CI, confidence interval; SE, standard error.

## Data Availability

The data that support the findings of this study are available from the corresponding author upon reasonable request.
